# Elevated wildlife-vehicle collision rates during the COVID-19 pandemic

**DOI:** 10.1038/s41598-021-99233-9

**Published:** 2021-10-14

**Authors:** Joel O. Abraham, Matthew A. Mumma

**Affiliations:** Department of Ecology and Evolutionary Biology, Princeton University, Princeton, NJ 08544 USA

**Keywords:** Behavioural ecology, Conservation biology, Environmental impact, Sustainability

## Abstract

Wildlife-vehicle collisions threaten both humans and wildlife, but we still lack information about the relationship between traffic volume and wildlife-vehicle collisions. The COVID-19 pandemic allowed us to investigate the effects of traffic volume on wildlife-vehicle collisions in the United States. We observed decreased traffic nationwide, particularly in densely populated states with low or high disease burdens. Despite reduced traffic, total collisions were unchanged; wildlife-vehicle collisions did decline at the start of the pandemic, but increased as the pandemic progressed, ultimately exceeding collisions in the previous year. As a result, nationwide collision rates were higher during the pandemic. We suggest that increased wildlife road use offsets the effects of decreased traffic volume on wildlife-vehicle collisions. Thus, decreased traffic volume will not always reduce wildlife-vehicle collisions.

## Introduction

Collisions between motor vehicles and wildlife pose a major problem globally, amounting to financial costs of more than 8 billion dollars (USD)^1^, resulting in an estimated 26,000 human injuries^2^, and causing countless wildlife fatalities annually in the United States alone^3^. These societal and ecological costs are only expected to grow as the number of vehicles on roads increases, road networks grow, and certain wildlife populations, primarily deer (family *Cervidae*), become more abundant and expand their distributions^1,4–6^: though wildlife populations of all sorts are negatively impacted by collisions with vehicles^3^, damage to vehicles and injury to drivers primarily result from collisions with large animals, particularly deer, and are the collisions most often reported to automobile insurance companies (Table S1)^3–5^. To mitigate the impacts of these costly collisions, a better understanding of the relationship between traffic volume and wildlife-vehicle collisions (WVCs) is essential.

The number of WVCs is a function of the number of vehicles on roads (hereafter, traffic volume) as well as the use of roads by wildlife^1,7^. Though road use by animals can simply be incidental^8,9^, some animals preferentially use roads^10–13^: roads and roadsides allow animals to move unincumbered throughout the landscape and can provide unique nutritional opportunities^10,11^. Their road use, in turn, puts wildlife at risk of being struck by automobiles^9–11^. As such, decreases in either traffic volume or wildlife road use—via changes in wildlife population abundance, the appeal of roads, or animal behavior—should cause concomitant declines in WVCs: if there are less cars on the road or less wildlife crossing roads, then the number of collisions should decrease^1,7^. Research suggests, however, that changes in traffic volume might impact the behavior of certain wildlife species, particularly large mammals, which might complicate the relationship between traffic volume and WVCs^3,14–16^. Because of the appeal of roads and roadsides to wildlife^10–13^, declines in traffic volume might reduce the perceived risk of using roads by wildlife, thereby leading to greater use of areas near roads and more frequent crossing of roads. Such changes in wildlife risk perception might counteract the effects of lower traffic volume on WVCs, potentially causing net increases in collision rates between vehicles and wildlife (WVCs/traffic volume)^3^, and resulting in a non-linear relationship between traffic volume and WVCs. Thus, potential changes in animal behavior create uncertainty regarding how changes in traffic volume affect the number and rate of WVCs.

The onset of the COVID-19 pandemic presents a natural experiment by which to examine the relationship between traffic volume and WVCs^17^. Restrictions implemented to mitigate the spread of the novel coronavirus SARS-CoV-2 caused substantive decreases in human mobility^18^, altogether resulting in dramatic declines in traffic volume globally during the first several months of the pandemic^17,19,20^. In the United States, these declines appear to have been greatest in states with the most severe restrictions^18,21^, suggesting that the restrictions themselves, but possibly also the associated fear of contracting SARS-CoV-2, were largely effective at reducing mobility^22–24^. Concurrently, changes to animal behavior were widely noted during the onset of the COVID-19 pandemic; within bird populations in the San Francisco Bay Area of California, for example, lower traffic volume resulted in less noise pollution, which caused songbirds to change their songs^20^. One of the most pervasive changes to animal behavior observed during the beginning of the pandemic was alterations in wildlife space-use^17,25^. There were widespread accounts of wildlife moving into urban and peri-urban spaces around the world and making increased use of newly vacant human spaces^25^. Although the aforementioned changes to wildlife space-use remain largely anecdotal^20^, such accounts suggest that wildlife may have increased their use of roads and areas near roads during the pandemic in response to decreases in traffic volume^17,25^. Thus, compensatory changes in animal behavior might have offset any effects of lower traffic volume on the frequency of WVCs during the pandemic, resulting in little net change in the number of WVCs.

**Figure 1:**
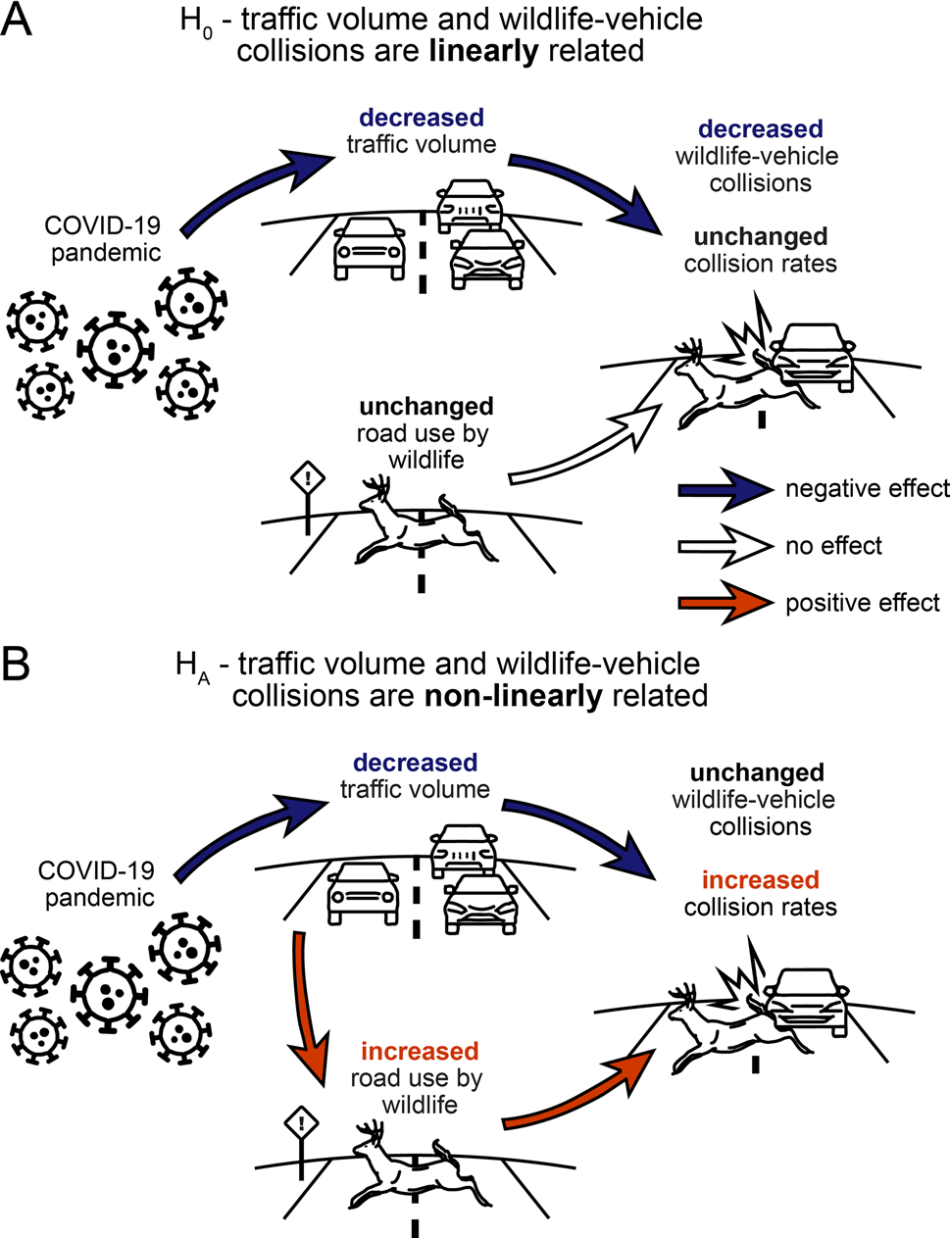
Potential relationships between traffic volume and wildlife-vehicle collisions. Substantial declines in human mobility have resulted from the COVID-19 pandemic, leading to declines in traffic volume on roads. (**A**) If the use of roads by wildlife is independent of traffic volume (H_0_), declines in traffic volume will reduce the number of wildlife-vehicle collisions and collision rates will remain unchanged, indicating a linear relationship between traffic volume and wildlife-vehicle collisions. Alternatively, (**B**) if declines in traffic volume reduce the perceived risk of roads by wildlife (H_A_), the number of wildlife-vehicle collisions will remain largely unchanged and collision rates will increase. As such, compensatory increases in wildlife road use in response to lower traffic volume might drive a non-linear relationship between traffic volume and wildlife-vehicle collisions. Arrow and bold text colors correspond to the direction of effects; blue corresponds to negative effects and decreases, red to positive effects and increases, and white arrows and bold black text to no effect and no change.

Given observations from the first several months of the pandemic indicating changes in human mobility and animal behavior, we hypothesized how the number and rate of WVCs in the United States might respond to changes in traffic volume during the COVID-19 pandemic (Fig. 1). If wildlife road use is independent of traffic volume (H_0_), then declines in traffic volume during the pandemic will cause a decrease in WVCs, whereas the rate of WVCs will remain unchanged, thus suggesting that wildlife use roads in a consistent manner regardless of changes in traffic volume (Fig. 1A)^1,7,26^. Furthermore, decreases in WVCs will be greatest where decreases in traffic are greatest, resulting in a positive relationship between changes in WVCs and changes in traffic volume. However, if wildlife road use is dependent in part on traffic volume (H_A_), then there will be no clear directional change in the overall number of WVCs, and collision rates between vehicles and wildlife will increase (Fig. 1B), thus suggesting that wildlife respond to declines in traffic volume by using roads more frequently^3,14–16,27–29^. Likewise, since compensatory changes in wildlife behavior should decouple WVCs from traffic volume, any changes in WVCs will be unrelated to changes in traffic, such that there will be no relationship between changes in WVCs and changes in traffic volume.

To evaluate our hypotheses, we analyzed data on traffic volume^30^ and WVCs (from animal-related car insurance claims^31^) across the United States, comparing the insurance year before the pandemic (July 2018–June 2019) and the insurance year including the first several months of the COVID-19 pandemic (July 2019–June 2020) to determine if there were directional changes in total WVCs and/or collision rates with the onset of the pandemic. Monthly insurance claim totals were available for the United States as a whole, whereas only annual insurance claim totals were available for individual states; as such, we were able to compare monthly trends in WVCs for the entire country and changes in WVCs between years for individual states. We also incorporated national and state-level data on human population densities^32^, SARS-CoV-2 disease burdens (the proportion of individuals with active SARS-CoV-2 infections)^33^, baseline traffic volume^30^, and the severity of COVID-19 restrictions (from a published synthesis^21^) (Table S2), to evaluate the contributions of these factors to observed changes in traffic volumes. Altogether this study presents novel evidence for compensatory road use by wildlife in response to decreased road traffic, and a non-linear relationship between traffic volume and WVCs.

## Results

**Figure 2:**
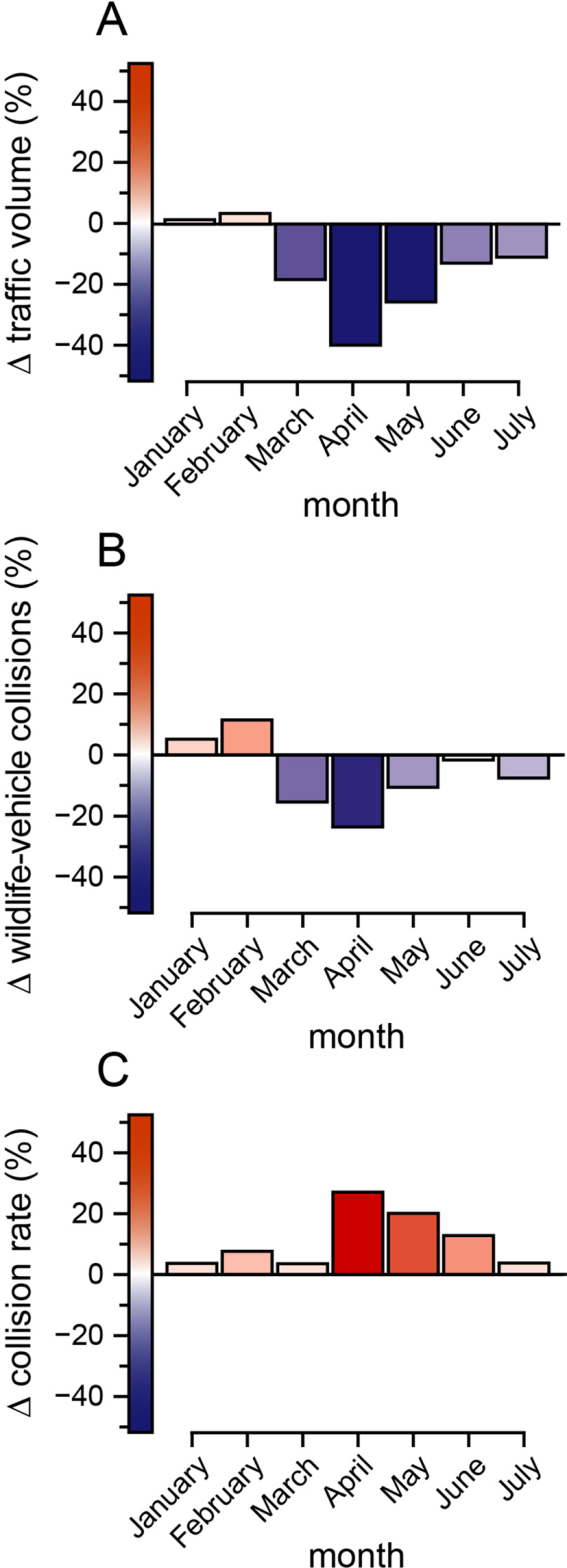
Monthly effects of the pandemic for the entire United States. Percent changes (Δ) in nationwide (**A**) traffic volumes (measured in millions of vehicle-miles traveled), (**B**) wildlife-vehicle collisions (the number of animal-related insurance claims), and (**C**) collision rates (the number of collisions per vehicle-miles traveled) between 2019 and 2020 for the months of January–July. Blue bars correspond to decreases and red to increases, with darker colors signifying greater relative changes.

**Figure 3:**
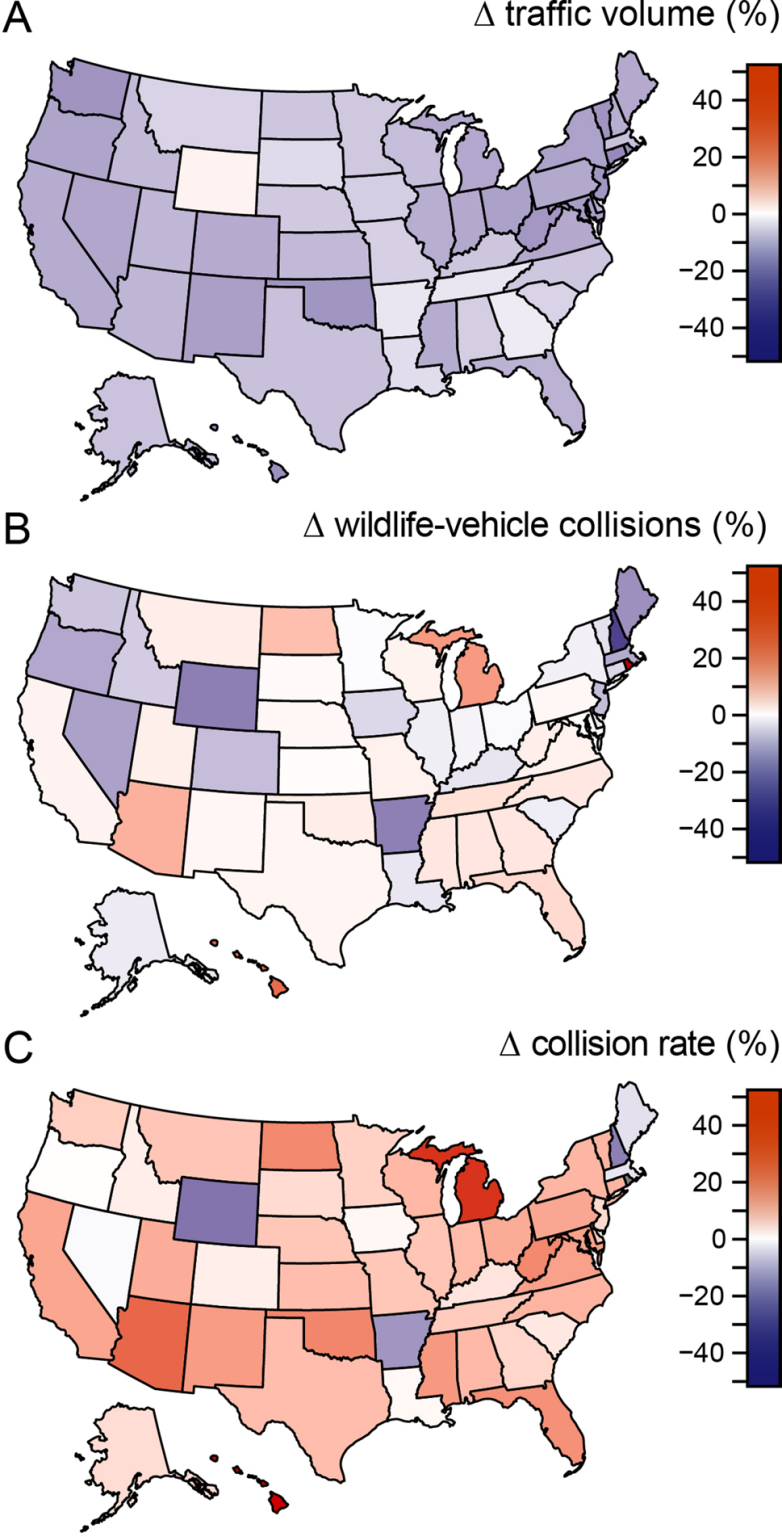
State-by-state effects of the pandemic across the United States. Percent changes (Δ) in (**A**) traffic volumes (millions of vehicle-miles traveled), (**B**) wildlife-vehicle collisions (number of animal-related insurance claims), and (**C**) collision rates (number of collisions per vehicle-mile traveled) between July 2018–June 2019 and July 2019–June 2020 for all fifty states and Washington, D.C. States colored blue experienced decreases and states colored red experienced increases, with darker colors signifying greater relative changes. Maps were generated in R 3.6.1^45^ with package ‘usmap’^49^.

**Figure 4:**
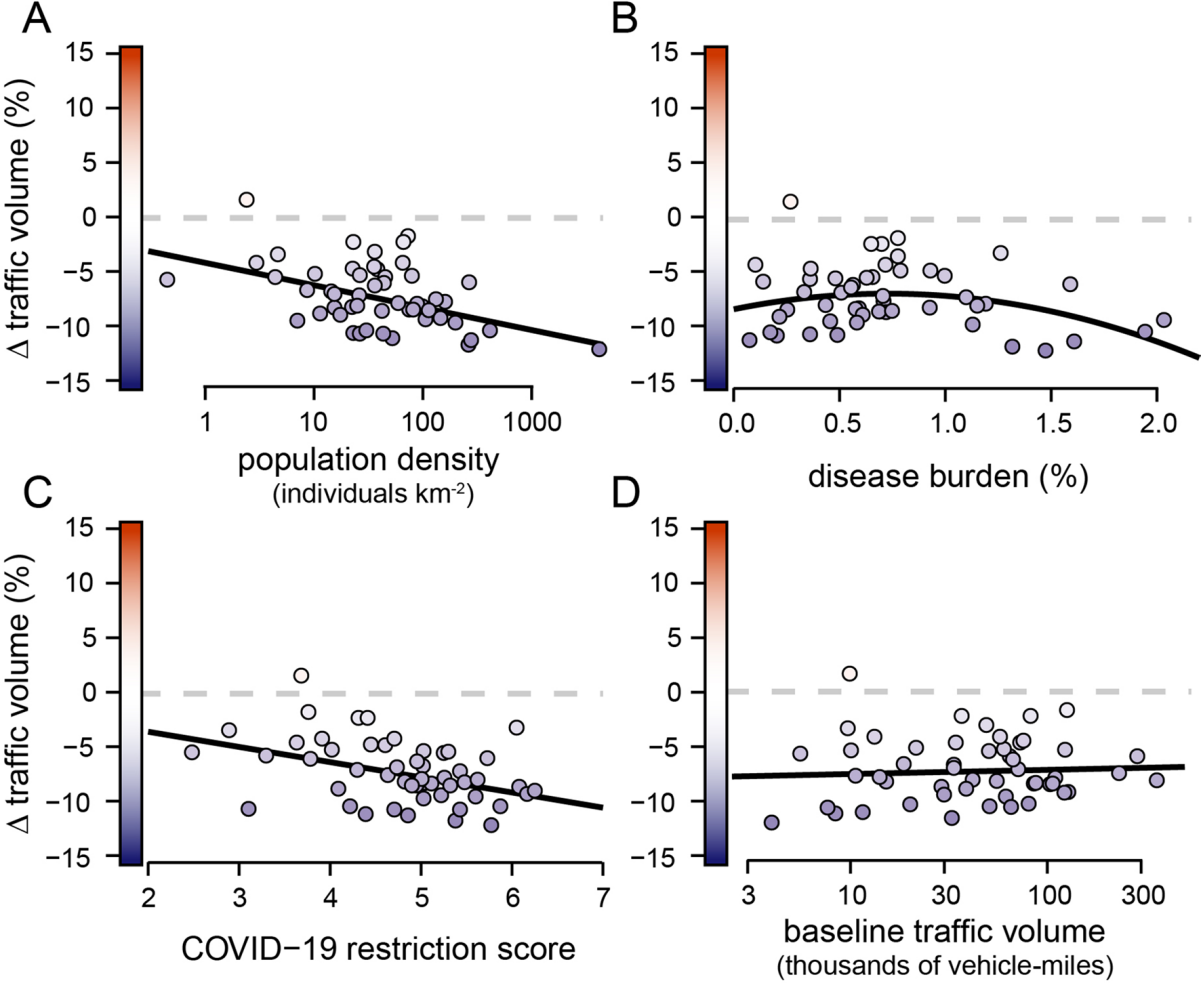
Drivers of changes (Δ) in traffic volume across the United States. Changes in traffic volume between July 2018–June 2019 and July 2019–June 2020 across the fifty states and Washington, D.C. were correlated with (**A**) human population density and (**B**) local disease burden: traffic volume declined most in densely populated states and in states with either low or high disease burdens. Both (**C**) the severity of COVID-19 restrictions and (D) baseline traffic volumes were also related to traffic volume: traffic volume decreased most where restrictions were most severe and decreased least in states with high baseline traffic volume. However, COVID-19 restrictions and baseline traffic volume were not significant in all of our top models (Table S4). Response curves were predicted using model 26, which included all four covariates as predictors of changes in traffic volume (Table S4). Blue points correspond to decreases and red to increases, with darker colors signifying greater relative changes.

Comparisons of traffic volumes across the United States between the insurance year preceding the pandemic (July 2018–June 2019) and the insurance year including the beginning of the pandemic (July 2019–June 2020) revealed a 7.2% reduction in traffic volume across the entire United States (Fig. 2A), consistent with other work documenting substantial declines in road traffic globally with the onset of the pandemic^10,12,13,20^. Reductions in annual traffic were ubiquitous across states (*t*_*50*_ = − 6.20, *P* < 0.05) (Fig. 3A) and were related to human population density and local disease burden (Table S4); traffic declined most in densely populated states, such as Connecticut and Rhode Island, and declines in traffic were greatest in states with either low or high disease burdens, including Hawai’i and New Jersey (Fig. 4). Both the severity of COVID-19 restrictions and baseline traffic volumes also appeared to influence annual traffic volume, with traffic decreasing most where restrictions were most severe and decreasing least in states with high baseline traffic (Fig. 4), although these effects were not significant in all of our top models (Table S4). Annual declines in traffic were driven by substantially lower traffic volumes March–June of 2020 (Fig. 2A). These dramatic reductions in monthly traffic were well beyond the bounds of normal variability in nationwide traffic volume (*F*_*1,77*_ = 21.99, *P* < 0.001) (Fig. S1). Monthly declines in traffic volume were not related to national disease burden (*t*_*2,4*_ = 2.220, *P* = 0.091) but were correlated with the severity of COVID-19 restrictions (*t*_*2,4*_ = -16.817, *P* < 0.0001) (Fig. S2), with a maximum decline of 39.7% in April 2020 when COVID-19 restrictions were most stringent (Fig. 2A).

**Figure 5:**
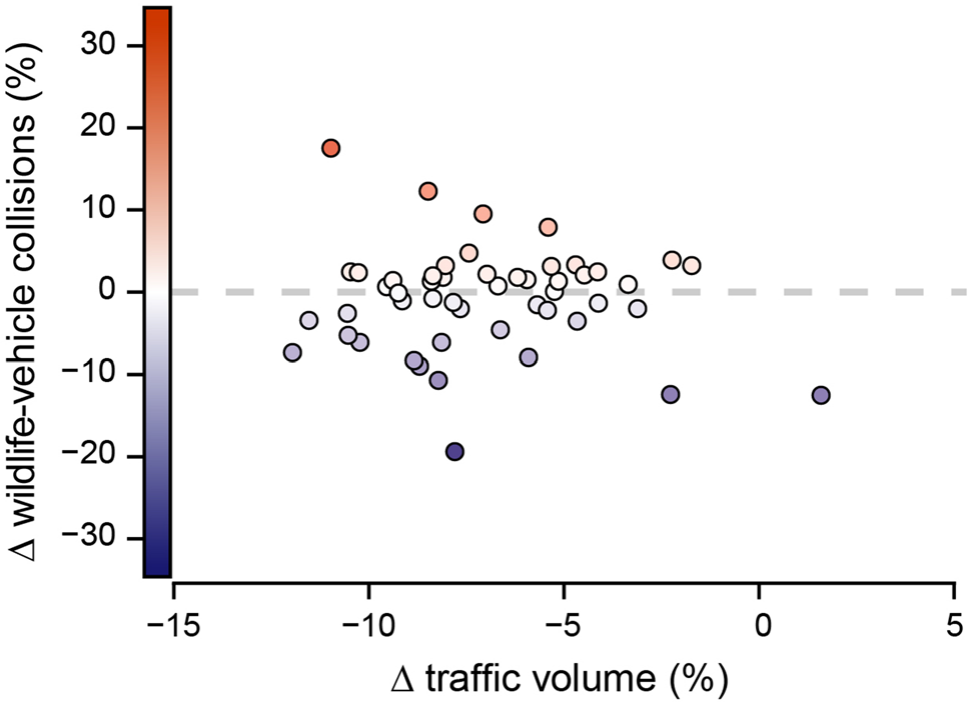
Relationship between changes (Δ) in traffic volume and changes in wildlife-vehicle collisions across the United States. Changes in traffic volume between July 2018–June 2019 and July 2019–June 2020 across the fifty states and Washington, D.C. were not predictive of concurrent changes in wildlife-vehicle collisions (*F*_*1,49*_ = 1.94, *P* = 0.17), consistent with the existence of compensatory effects of wildlife road use in response to lower traffic volume. Blue points correspond to decreases and red to increases, with darker colors signifying greater relative changes.

In notable contrast to the widespread declines in traffic volume, the number of WVCs was largely unchanged between the pre-pandemic and pandemic insurance years, consistent with H_A_ (Fig. 1B). Comparisons of animal-related car insurance claims—the most robust estimate of WVCs at such large spatial scales^1,34^—revealed that the number of WVCs actually increased by 0.5% (from 2,088,263 to 2,098,533) between the two insurance years, though this increase was not significant (*t*_*6*_ = − 1.34, *P* = 0.23). The number of WVCs did decline in the first several months of the pandemic, peaking at a 23.3% decline in April, but started to return to baseline levels by June despite traffic volume remaining lower relative to the previous year (Fig. 2B). Nationwide patterns were spatially heterogeneous, with some states (like Michigan and North Dakota) experiencing increases in the number of WVCs and others (like New Hampshire and Wyoming) experiencing decreases, such that there was no clear directional trend across states (*t*_*50*_ = 0.58, *P* = 0.56) (Fig. 3B). Annual changes in the number of WVCs across states were unrelated to changes in traffic volume (*F*_*1,49*_ = 1.94, *P* = 0.17) (Fig. 5), a pattern that is likewise consistent with the hypothesis that traffic volume affects animal behavior (H_A_), and thereby decouples changes in traffic volume from changes in WVCs.

Comparisons of the rates of WVCs before and during the beginning of the pandemic were also consistent with the hypothesis that traffic volume influences how much wildlife use roads and roadsides (H_A_). Collision rates were 8.3% higher during the pandemic insurance year relative to the previous insurance year (Fig. 2C) (*t*_*6*_ = 3.57, *P* < 0.05). Consistent with the fact that WVCs are increasing through time^1,4–6^, collision rates were elevated in the months before the pandemic began as compared to the previous year but peaked after the onset of the pandemic with a dramatic 27.2% increase in April (Fig. 2C). Increases in collision rates persisted through July despite traffic volume once again approaching baseline levels (Fig. 2C). The observed increases in collision rates during the pandemic year were nearly ubiquitous across states (*t*_*50*_ = 4.24, *P* < 0.0001); only six states (Arkansas, Massachusetts, Maine, Nevada, New Hampshire, and Wyoming) saw collision rates decline during the pandemic year (Fig. 3C).

## Discussion

Altogether, we found that, while traffic volume declined by > 7% during the pandemic year (with a maximum monthly decline of nearly 40%), the absolute number of annual WVCs was largely unchanged. This resulted in significant increases of > 8% in collision rates between vehicles and wildlife during the pandemic year, peaking at a > 27% nationwide increase in April 2020. Other studies from the first several months of the pandemic documented similar transient declines in the number of WVCs when the pandemic began which then reversed in many jurisdictions as the pandemic progressed and traffic rebounded^26,27^. We observed a similar pattern over the first five months of the pandemic at the national scale (Fig. 2): WVCs initially declined during the pandemic in step with declines in traffic volume, but then started to increase to baseline levels at a faster rate than traffic, possibly due to behavioral lags by wildlife following traffic-mediated increases in wildlife road use. Though based on coarse-scale data, our research aligns with assertions from studies during^27^ and prior to the pandemic^3,15,16,28,29^ that the relationship between traffic volume and WVCs is non-linear.

We postulate that the observed non-linear relationship between traffic volume and WVCs is the result of greater use of roads and roadsides by certain wildlife species, namely large mammals (Table S1), in response to decreasing traffic volume, as prior research has suggested^3,14–16^. This explanation is consistent with accounts of various wildlife species making increased use of human spaces during the pandemic^17,20,21^: with less cars on the roads, wildlife might be less deterred from roads by the noise and light pollution that accompany high traffic volumes^9–11,20^ and perceive roads as less risky, thereby increasing their willingness to attempt road crossings^3,8,15,16^. Beyond incidentally crossing roads while moving about the landscape^8,9^, wildlife might be attracted to roads for travel, mates, or other resources^8,10,11^. Many animals are shown to utilize roads to move efficiently across the landscape^11,12^, and roads and the surrounding areas are comparatively open, such that wildlife might select roads and roadsides for enhanced visibility to find mates, detect predators, or locate prey^10,13^. Roadsides also can provide foraging opportunities and essential nutrients for wildlife via abundant, high-quality early successional vegetation and high salt concentrations^10,11^. As such, decreased road traffic during the pandemic might have caused certain wildlife species to tolerate the risks associated with roads in order to access the benefits of roads and roadsides.

An alternative explanation for the observed increases in collision rates is that human driving behavior, rather than animal behavior, changed during the pandemic. With fewer cars on the road, people might drive faster^35^, rendering it more difficult for both humans and wildlife to avoid collisions^3^. Preliminary studies from throughout the United States have indeed suggested changes to human driving behavior during the pandemic, with several jurisdictions reporting increased vehicle speeds^35,36^. Despite reported increases in vehicle speeds, however, the total number of vehicle collisions (the sum of both wildlife and non-wildlife collisions) mirrored trends in traffic volume and declined considerably during the pandemic^37,38^. Thus, because changes to human behavior appear to have had a minimal effect on vehicle collisions overall, it is unlikely that the observed changes in collision rates are due to increased vehicle speeds alone. Still, we cannot discount the possibility that changes to human driving behavior contributed to the patterns documented here, and future work should more explicitly test the relative effects of changes in traffic volume on both human driving behavior and wildlife space-use, as well as the resultant impacts on WVCs.

A greater understanding of human driving behavior would also help explain our findings regarding changes in traffic patterns during the pandemic. Nationwide, the severity of COVID-19 restrictions accounted for a large amount of the variation in changes in monthly traffic volume (*R*^2^ = 0.968), but the severity of restrictions was less influential on changes in yearly traffic across states (Tables S3 and S4). Restrictions implemented throughout the pandemic were largely enacted for the purpose of minimizing travel, and other research has demonstrated that these restrictions were effective at reducing human mobility^18,21^. Our state-level findings, however, imply that it was not only the restrictions themselves that reduced travel, but possibly also the associated anxiety regarding the risk of contracting the SARS-CoV-2 virus, as has been suggested in other studies^21–24^; although we observed the greatest declines in traffic volume early in the pandemic (Fig. 2A) when restrictions were most stringent (Fig. S2)^21^, there was widespread anxiety about the risks posed by SARS-CoV-2 during this time^22,23^, which likely motivated people to stay home independent of restrictions^24^. Indeed, anxiety and risk perception might explain the relationship between traffic volume and the other covariates in our top models (Table S4). Declines in traffic were greatest in the most densely populated states (Fig. 4A) and in states that had the highest and the lowest disease burdens (Fig. 4B). The risk of SARS-CoV-2 transmission is greater in more densely populated states due to the close proximity of and frequent interactions amongst people^21^. As such, people may have altered their road use more in densely populated states as compared to sparsely populated ones due to differing perceptions of disease transmission risk^23^—though differences in infrastructure in relation to population density likely contributed to this pattern as well^39^. Similarly, declines in traffic volume in states with larger outbreaks of SARS-CoV-2 might have been driven by increases in the perceived risk of contracting the virus^21,23^. Alternatively, traffic reductions in states with low disease burdens might reflect increased compliance with stay-at-home orders, and therefore less opportunity for disease spread^40,41^; essentially, reductions in traffic volume might be the cause of locally low disease burdens therein, rather than a consequence. Altogether, we posit that the observed heterogeneity in traffic volume between states is, at least in part, attributed to differences in the perceived risk posed by the SARS-CoV-2 virus.

Regardless of the mechanisms underlying changes in traffic volume and WVCs, our observation that the annual number of WVCs was largely unchanged despite substantive declines in traffic volume has implications for mitigating WVCs going forward. Most directly, the lack of a directional change in WVCs suggests that road traffic levels in the United States are currently such that even large decreases in traffic volume would have minimal long-term effects on the absolute number of WVCs. As such, decreasing collisions by reducing traffic volume would require even larger and longer-lasting changes in traffic than those observed during the pandemic. Since such massive and sustained reductions in traffic are unlikely^4–6^, WVCs in the United States essentially represent a fixed cost as of now, both for human society and wildlife populations. As such, these transient decreases in traffic likely provided minimal reprieve to large mammals from collision-induced mortality, in contrast to speculation that changes in human mobility during the COVID-19 pandemic had substantial positive effects for wildlife populations by freeing wildlife from the pervasive direct and indirect effects of humans^17–20,26,27,42^.

Indeed, it is possible that short-term decreases in traffic volume might ultimately be harmful to those wildlife species that increased their road use. Although the increases in collision rates we observed at the beginning of the pandemic were rapid and corresponded to nationwide declines in traffic volume (see also^26,27^), collision rates remained elevated even as traffic approached baseline levels in July (Fig. 2B). If wildlife responses to changes in traffic are asymmetric (i.e*.,* increases in wildlife road use following declines in traffic occur more rapidly than decreases in wildlife road use in response to increased traffic), then short-term declines in traffic volume might lead to net increases in the number WVCs over longer timeframes, ultimately proving detrimental to certain wildlife populations^1,3^. Future work should evaluate the long-term effects of the pandemic on wildlife populations, specifically with regards to collision-induced mortality^17,20,26,27,42^.

Although the COVID-19 pandemic provided an opportunity to examine the short-term effects of transient decreases in traffic volume on WVCs, the longer-term effects of expanding human populations, greater road densities, and altogether higher traffic volumes on WVCs are less clear. Similar to the increases in wildlife road use in response to decreases in traffic volume theorized here, steady increases in traffic might reduce wildlife road use long-term^3,14–16^; since road traffic is indeed increasing through time^4–6^, we might therefore see declines in WVCs as roads become more effective at repelling wildlife^1,3,14^. Although these reductions in vehicle-induced wildlife mortality are welcome, this would see roads increasingly serve as barriers to animal movement and gene flow^43^, further fragmenting already disconnected wildlife populations^8^. Thus, policy makers and urban planners should invest in infrastructure such as overpasses, underpasses, and fencing that enables wildlife to cross high-traffic roads safely or directs wildlife towards low-risk areas^8,9^. Even substantive short-term declines in road traffic are not sufficient to mitigate wildlife-vehicle conflict on their own.

## Methods

### Data sources

Our primary motivation for this study was to determine the effects of the pandemic on WVCs throughout the United States. As such, we first sought out data on WVCs for the United States. For the purposes of this study, we used animal-related automobile insurance claims data as a proxy for WVCs; insurance industry claims are one of the main data sources used to investigate national patterns of WVCs in the United States, as other nationwide databases do not exist^1,26^. These data are likely underestimates of total WVCs, as only about half of all WVCs are reported to insurance companies^34^. The data should, however, accurately reflect relative changes in WVCs given that data collection methods are consistent across years^1,34^. Also, insurance claim data largely reflect collisions with large-bodied animals, particularly deer and other large mammals (Table S1), as these collisions result in damage to vehicles and injuries to drivers and are therefore principally reported to automobile insurance companies^1,3^. As such, the patterns observed here reflect the behavior of large mammals specifically (Table S1).

We procured the data on animal-related automobile insurance claims for this study from State Farm Mutual Automotive Insurance Company (hereafter, State Farm)^31^. State Farm estimates industry-wide insurance claims from their own claim numbers^44^; total industry-wide claims are computed by scaling up their individual claim data using their market penetration (i.e., State Farm’s proportion of the total number of insured drivers)^1^. These industry-wide estimates are then validated with collision data maintained by the highway service^44^. The insurance claim year begins in July and ends in June; because State Farm changed their claim reporting methodologies in 2018^44^, data were only available starting July 2018. State Farm computes state-level insurance claim totals for the entire insurance year only (July–June), whereas they calculate national insurance claim totals for each month. As such, annual wildlife-vehicle collision data were available for each state and Washington D.C. for July 2018–June 2019 and for July 2019–June 2020, whereas monthly insurance claim estimates were available at the national scale for July 2018–July 2020. While these data restricted us to annual comparisons for individual states, the period of July 2019–June 2020 consists largely of the COVID-19 pandemic, including the critical first several months. Also, the fact that yearly totals include claims from before the onset of the pandemic means that any changes reported here are underestimates, and that the changes are therefore of an even greater magnitude than we capture.

We then assembled corresponding road traffic data from the United States Federal Highway Administration^30^, to determine the effect of the pandemic on traffic volume in the Unites States. The Federal Highway Administration tabulates monthly reports of vehicle traffic based on hourly traffic count data reported by each state and Washington, D.C.^30^. The hourly traffic data are collected at approximately 5000 continuous traffic counting locations nationwide and aggregated into monthly estimates per state. These monthly estimates are re-adjusted annually to correspond to the vehicle-miles of travel from the Highway Performance Monitoring System and are continually updated with additional data. We collected monthly data on national traffic volume (i.e., total road traffic for the entire United States) from January 2015–July 2020, to evaluate if traffic levels during the pandemic were within the bounds of normal annual variation in traffic volume (Fig. S2). Then, to match the scale of our WVC data, we assembled data on annual traffic volumes for each state and Washington D.C. from July 2018–June 2019 and July 2019–June 2020.

To evaluate if human population density might have contributed to observed changes in traffic volume, we gathered 2019 population size estimates for each individual state and Washington D.C. Data were derived from the United States Census Bureau^32^. The Census Bureau produces estimates of the total resident population size for every state on an annual basis. These estimates are extrapolations from the most recent census, which occurred in 2010, using an administrative record-based component of change method, which updates the latest census population size using data on births, deaths, and domestic and international migration^32^. We used 2019 estimates of population size because data from the 2020 census were not available, as the 2020 census was still being conducted at the time of the analyses. To convert population size to population density, population data were divided by the corresponding area (km^2^) of each state and Washington D.C.

We anticipated that the magnitude of changes in traffic volume might be determined by the severity of COVID-19 restrictions, as other research suggested that states with more severe restrictions should exhibit greater reductions in human mobility^18,21^. As such, we used a published policy database of non-pharmaceutical interventions to calculate restriction scores^21^. Daily data on the severity of restrictions were available for each state. Thus, we calculated a monthly score for the whole United States by averaging daily values in a given month across all states and Washington D.C. We then calculated restriction scores for each state by averaging all the values between March 1st (when restrictions were first put into effect) and June 30th (the last insurance claim data included in annual estimates of total animal-related insurance claims for each state). Table S2 contains an explanation of the interventions and mitigation measures that correspond to each score.

Finally, we wanted to evaluate if changes in traffic volume across the United States were related to the local severity of the pandemic. States with larger outbreaks of SARS-CoV-2 might be expected to exhibit greater reductions in traffic, due to increases in the perceived risk of contracting SARS-CoV-2 therein^21,23^. Alternatively, the greatest reductions in traffic might be observed in states with low disease burdens, as reductions in traffic might in fact reflect increased compliance with stay-at-home orders, and therefore less opportunity for disease spread^40,41^. Finally, we speculated that both these dynamics could be occurring simultaneously, driving a non-linear relationship between disease burden and reductions in traffic volume. We therefore calculated disease burden for every state; we gathered data on the cumulative number of SARS-CoV-2 cases reported through June 30th, 2020 for each state from the COVID Tracking Project^33^ and standardized these data by total state population to compute the proportion of the total state population afflicted by SARS-CoV-2, which we defined as the local disease burden.

### Data analyses

Data analyses were performed using R 3.6.1^45^. To determine if there were clear changes during the initial months of the pandemic, we performed paired *t* tests to compare monthly data (January–July) between 2019 and 2020. To evaluate if reductions in monthly traffic were within the bounds of normal variation in traffic volume, we performed paired *t* tests to compare monthly traffic volumes in the years prior to and during the pandemic (Fig. S2). To determine whether changes were consistent across the United States, we also used paired *t* tests to compare annual data for all fifty states and Washington D.C. between insurance claim years (July 2018–June 2019 and July 2019–June 2020) (Fig. 3).

Next, we sought to understand the drivers of the observed changes. First, we modeled if monthly reductions in national traffic volumes were related to the severity of COVID-19 restrictions and national disease burden using linear regression (Fig. S2). We then used model selection to evaluate potential drivers of observed changes in traffic volume across the United States (Table S3). We built a series of linear models predicting changes in traffic volumes in each of the fifty states and Washington D.C. and compared AIC_c_ values to determine the most parsimonious models using the package ‘MuMIn’^46^. Predictors were baseline road traffic volume (i.e., traffic volumes in July 2018–June 2019), state population densities, the severity of COVID-19 restrictions, and local disease burden (Fig. 4); both baseline traffic volume and human population density were log-transformed to meet assumptions of normality. We also included interactions between variables in our model set to account for potential colinear relationships among our predictors (see Table S3). Coefficients from the top models (ΔAIC_c_ < 2)^47^ are included in Table S4. We visually confirmed that all top models met the assumptions of linear modeling from model diagnostic plots (Fig. S3) and further validated that all assumptions were satisfied using the package ‘olsrr’^48^. Finally, to determine if there was a relationship between changes in traffic and changes in WVCs for each of the states within the United States, we fitted a linear model, with changes in traffic volume across years for each state as the independent variable and changes in total WVCs across years for each state as the dependent variable (Fig. 5).

## Supplementary Information

Supplementary Information.
