# Supplementary material for: Elevated wildlife-vehicle collision rates during the COVID-19 pandemic
Source: Sci Rep. 2021 Oct 14;11:20391. doi: 10.1038/s41598-021-99233-9 (PMC8516972; doi:10.1038/s41598-021-99233-9)
Supplement: Supplementary file 1 — Supplementary Information. [file 41598_2021_99233_MOESM1_ESM.docx]

**SUPPLEMENTARY MATERIALS**

**MANUSCRIPT TITLE:** Elevated wildlife-vehicle collision rates during the COVID-19 pandemic

**AUTHORS:** Joel O. Abraham^1*^ and Matthew A. Mumma^1^

**AFFILIATIONS:**

^1^Department of Ecology and Evolutionary Biology, Princeton University, Princeton, NJ, 08544

*Corresponding author – email: joeloa@princeton.edu, phone: +1 (609) 258-5810, address: Department of Ecology and Evolutionary Biology, Princeton University, Princeton, NJ, 08544

**TABLES AND FIGURES**

**Table S1. Animal taxa reported in animal-related insurance claims.** Numbers reflect the sum total of all claims for each animal across all fifty states and Washington, D.C. reported in July 2018–June 2019 and July 2019–June 2020. Animal names are shown as reported to State Farm.

| **Animal (as reported to State Farm)** | **July 2018**–**June 2019** | **July 2019**–**June 2020** |
| --- | --- | --- |
| Deer | 1,151,968 | 1,345,318 |
| Dog | 87,038 | 90,324 |
| Rodents | 80,969 | 93,741 |
| Raccoon | 49,177 | 60,238 |
| Cattle | 42,992 | 43,044 |
| Coyote | 17,453 | 19,590 |
| Turkey | 16,502 | 15,391 |
| Bird | 13,083 | 14,395 |
| Cat | 12,675 | 13,967 |
| Bear | 11,877 | 12,469 |
| Squirrel | 11,377 | 13,307 |
| Pig | 10,329 | 12,268 |
| Horse | 9,409 | 9,401 |
| Fox | 4,686 | 5,067 |
| Elk | 4,625 | 5,024 |
| Buzzard | 4,601 | 4,749 |
| Geese | 3,492 | 3,661 |
| Rabbit | 3,364 | 4,053 |
| Possum | 3,096 | 3,460 |
| Eagle | 2,486 | 2,928 |
| Pheasant | 2,389 | 2,586 |
| Moose | 2,310 | 2,524 |
| Duck | 2,279 | 2,072 |
| Armadillo | 2,188 | 2,329 |
| Wolf | 1,999 | 2,066 |
| Beaver | 1,895 | 2,029 |
| Owl | 1,883 | 1,962 |
| Bobcat | 1,755 | 1,742 |
| Hawk | 1,755 | 1,993 |
| Chicken | 1,749 | 1,883 |
| Bat | 1,688 | 1,614 |
| Crane | 1,530 | 1,363 |
| Antelope | 1,286 | 1,369 |
| Crow | 1,079 | 1,284 |
| Turtle | 908 | 825 |
| Boar | 810 | 929 |
| Opossum | 768 | 892 |
| Groundhog | 640 | 611 |
| Alligator | 609 | 660 |
| Skunk | 609 | 538 |
| Goat | 573 | 746 |
| Chipmunk | 530 | 715 |
| Porcupine | 439 | 471 |
| Falcon | 402 | 403 |
| Sheep | 317 | 410 |
| Donkey | 305 | 330 |
| Seagull | 165 | 141 |
| Caribou | 116 | 86 |
| Reindeer | 18 | 24 |
| Tortoise | 6 | 18 |
| Unidentified animal | 514,065 | 291,522 |
| **TOTAL** | **2,088,263** | **2,098,533** |

**Table S2.** **Explanation of COVID-19 restriction severity scores and corresponding interventions.** Data on daily restrictions for each state were derived from Korevaar *et al.* (2020).

| **severity of restrictions** | **description of measures put in place to prevent the spread of COVID-19** |
| --- | --- |
| **1** | all businesses open with no restrictions, but state of emergency/public health emergency declared |
| **2** | all businesses open with some restrictions; PSAs regarding hygiene, social distancing, and limiting travel; quarantine required for out-of-state travelers; bans on gatherings greater than 1,000 individuals |
| **3** | bans on gathering of more than 250 people; partial school closures |
| **4** | bans on gatherings of more than 100 people; complete school or university closures; partial shelter-in-place or stay-at-home order |
| **5** | partial bars and restaurant closures (capacity limits put in place); bans on gatherings of more than 25–50 people |
| **6** | complete bar and restaurant closures, along with closures of other public spaces; bans on gatherings of more than 10 people |
| **7** | local shelter-in-place or stay-at-home orders in effect (affecting a substantial portion of state population but not the entire state) |
| **8** | statewide shelter-in-place or stay-at-home order in effect |

**Table S3. Model selection results for models predicting changes (Δ) in traffic volume.** The model with the lowest AIC_c_ included log(population density) and disease burden modeled as a second-degree polynomial (disease burden^2^). However, several other models had ΔAIC_c_ < 2 and variously included baseline traffic volume, the severity of COVID-19 restrictions, and disease burden (modeled linearly) as additional predictors.

| model number | model formula | *R*^2^ | *K* | logLik | AIC_c_ | ΔAIC_c_ | weight |
| --- | --- | --- | --- | --- | --- | --- | --- |
| 22 | **Δtraffic ~ disease burden^2^+ log(population density)** | **0.311** | **4** | **-115.83** | **243.00** | **0.00** | **0.182** |
| 26 | **Δtraffic ~ baseline traffic + log(population density) + disease burden^2^ + COVID-19 restrictions** | **0.379** | **6** | **-113.20** | **243.01** | **0.01** | **0.181** |
| 23 | **Δtraffic ~ COVID-19 restrictions + disease burden^2^+ log(population density)** | **0.341** | **5** | **-114.68** | **243.28** | **0.28** | **0.158** |
| 01 | **Δtraffic ~ baseline traffic + log(population density) + disease burden + COVID-19 restrictions** | **0.341** | **5** | **-114.70** | **243.30** | **0.30** | **0.156** |
| 04 | Δtraffic ~ log(population density) | 0.211 | 2 | -119.29 | 245.09 | 2.09 | 0.064 |
| 08 | Δtraffic ~ COVID-19 restrictions + log(population density) | 0.245 | 3 | -118.17 | 245.21 | 2.21 | 0.060 |
| 13 | Δtraffic ~ COVID-19 restrictions + disease burden + log(population density) | 0.271 | 4 | -117.26 | 245.86 | 2.86 | 0.044 |
| 10 | Δtraffic ~ disease burden + log(population density) | 0.229 | 3 | -118.71 | 246.29 | 3.29 | 0.035 |
| 14 | Δtraffic ~ COVID-19 restrictions * disease burden + log(population density) | 0.287 | 5 | -116.69 | 247.29 | 4.30 | 0.021 |
| 09 | Δtraffic ~ COVID-19 restrictions * log(population density) | 0.248 | 4 | -118.08 | 247.49 | 4.49 | 0.019 |
| 16 | Δtraffic ~ baseline traffic + COVID-19 restrictions | 0.210 | 3 | -119.33 | 247.54 | 4.54 | 0.019 |
| 07 | Δtraffic ~ COVID-19 restrictions | 0.171 | 2 | -120.56 | 247.64 | 4.64 | 0.018 |
| 15 | Δtraffic ~ (COVID-19 restrictions + log(population density)) * disease burden | 0.309 | 6 | -115.90 | 248.40 | 5.40 | 0.012 |
| 17 | Δtraffic ~ baseline traffic * COVID-19 restrictions | 0.221 | 4 | -118.96 | 249.26 | 6.26 | 0.008 |
| 19 | Δtraffic ~ baseline traffic + COVID-19 restrictions + disease burden | 0.210 | 4 | -119.33 | 250.00 | 7.00 | 0.006 |
| 11 | Δtraffic ~ COVID-19 restrictions + disease burden | 0.171 | 3 | -120.56 | 250.00 | 7.00 | 0.005 |
| 25 | Δtraffic ~ baseline traffic + COVID-19 restrictions + disease burden^2^ | 0.239 | 5 | -118.38 | 250.67 | 7.67 | 0.004 |
| 12 | Δtraffic ~ COVID-19 restrictions * disease burden | 0.177 | 4 | -120.37 | 252.08 | 9.08 | 0.002 |
| 20 | Δtraffic ~ baseline traffic + COVID-19 restrictions * disease burden | 0.212 | 5 | -119.25 | 252.40 | 9.41 | 0.002 |
| 21 | Δtraffic ~ (baseline traffic + COVID-19 restrictions) * disease burden | 0.243 | 6 | -118.24 | 253.09 | 10.09 | 0.001 |
| 06 | Δtraffic ~ disease burden^2^ | 0.098 | 3 | -122.70 | 254.28 | 11.28 | 0.001 |
| 02 | Intercept only | 0.000 | 1 | -125.34 | 254.92 | 11.92 | 0.000 |
| 05 | Δtraffic ~ disease burden | 0.035 | 2 | -124.42 | 255.35 | 12.36 | 0.000 |
| 24 | Δtraffic ~ baseline traffic + disease burden^2^ | 0.098 | 4 | -122.69 | 256.72 | 13.72 | 0.000 |
| 03 | Δtraffic ~ baseline traffic | 0.004 | 2 | -125.25 | 257.00 | 14.00 | 0.000 |
| 18 | Δtraffic ~ baseline traffic + disease burden | 0.044 | 3 | -124.20 | 257.26 | 14.26 | 0.000 |

**Table S4. Top (most parsimonious) models predicting changes in traffic volume.** Model coefficients, standard errors (SE), *t*-values, *P*-values, and variance inflation factors (VIF) for predictors of all models with ΔAIC_c_ < 2. Predictors shown in **bold** had a clear directional effect on changes in traffic volume.

|  | Coefficient | SE | *t*-value | *P*-value | VIF* |
| --- | --- | --- | --- | --- | --- |
| Model 22 | | | | | |
| **Intercept** | -5.970 | 1.199 | -4.980 | **> 0.001** | NA |
| **log(population density)** | -2.645 | 0.694 | -3.813 | **> 0.001** | 1.296 |
| **disease burden** | 703.600 | 269.8 | 2.608 | **0.012** | 1.138 |
| **(disease burden)^2^** | -3.080×10^4^ | 1.300×10^4^ | -2.369 | **0.022** | 1.138 |
| Model 26 | | | | | |
| Intercept | -3.970 | 2.203 | -1.802 | 0.078 | NA |
| baseline traffic | 0.601 | 0.366 | 1.640 | 0.108 | 1.126 |
| **log(population density** | -2.390 | 0.751 | -3.183 | **0.003** | 1.445 |
| **disease burden** | 567.600 | 271.500 | 2.091 | **0.042** | 1.178 |
| disease burden^2^ | -2.200×10^4^ | 1.330×10^4^ | -1.647 | 0.106 | 1.178 |
| **COVID-19 restrictions** | -0.896 | 0.497 | -1.803 | **0.078** | 1.269 |
| Model 23 | | | | | |
| Intercept | -3.269 | 2.201 | -1.486 | 0.144 | NA |
| **log(population density)** | -2.188 | 0.754 | -2.901 | **0.006** | 1.425 |
| COVID-19 restrictions | -0.719 | 0.494 | -1.456 | 0.152 | 1.239 |
| **disease burden** | 682.900 | 267.100 | 2.557 | **0.014** | 1.149 |
| **disease burden^2^** | -2.860×10^4^ | 1.290×10^4^ | -2.212 | **0.032** | 1.149 |
| Model 01 | | | | | |
| Intercept | -2.660 | 2.092 | -1.271 | 0.210 | NA |
| **baseline traffic** | 0.784 | 0.355 | 2.206 | **0.032** | 1.151 |
| **log(population density)** | -2.311 | 0.763 | -3.028 | **0.004** | 2.079 |
| disease burden | 149.900 | 98.900 | 1.516 | 0.136 | 1.715 |
| **COVID-19 restrictions** | -1.039 | 0.499 | -2.084 | **0.0423** | 1.562 |

*VIF < 5 indicates that multicollinearity of predictors is low


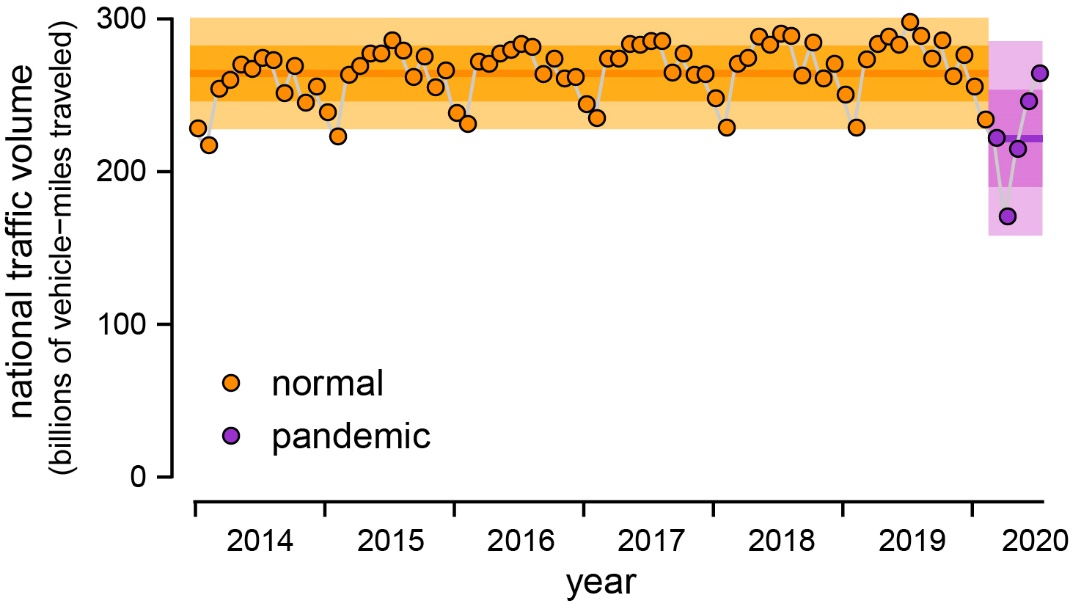


**Figure S1. Monthly patterns of nationwide traffic volume for the United States from 2015–2020**. Traffic volume during the COVID-19 pandemic was outside the bounds of normal variation in traffic volume (*F_1,77_* = 21.99, *P* < 0.001). *Orange* points correspond to monthly traffic volume pre-pandemic and *purple* points to monthly traffic volume during the first five months of the pandemic in the United States. *Dark orange* and *dark purple* lines correspond to pre-pandemic and pandemic means, respectively. The corresponding lighter *orange and purple* rectangles represent standard deviations from the means.


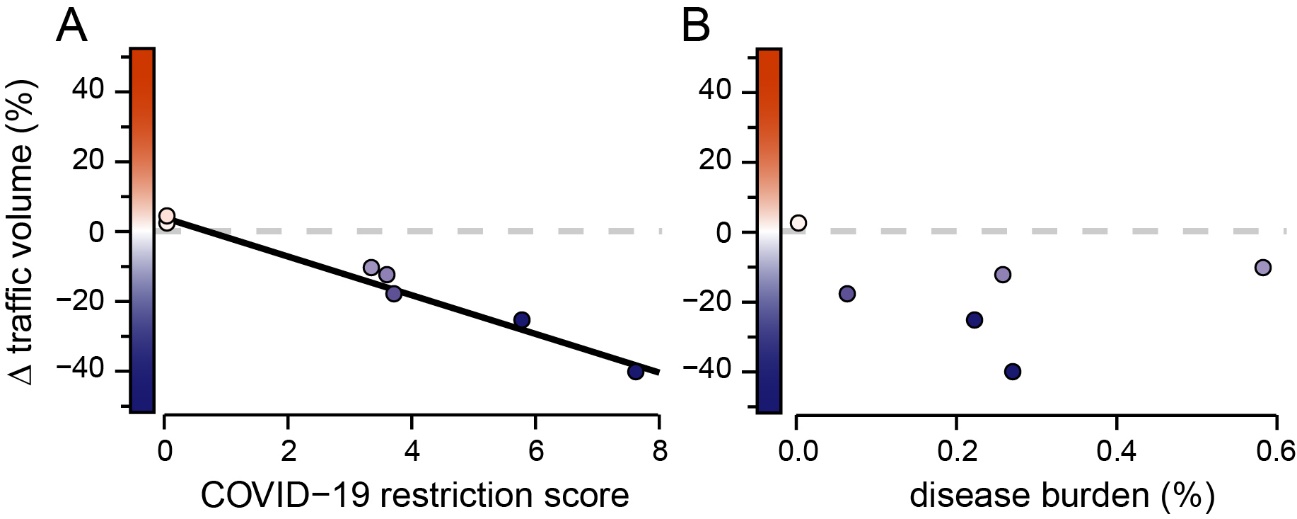


**Figure S2. Drivers of monthly changes (Δ) in traffic volume across the United States.** (A) Reductions in national traffic volume were greatest when COVID-19 restrictions were most severe (*t_2,4_* = -16.817, *P* < 0.0001), but (B) were not related to national disease burden (*t_2,4_* = 2.220, *P* = 0.091). *Blue* points correspond to decreases and *red* to increases, with darker colors signifying greater relative changes.


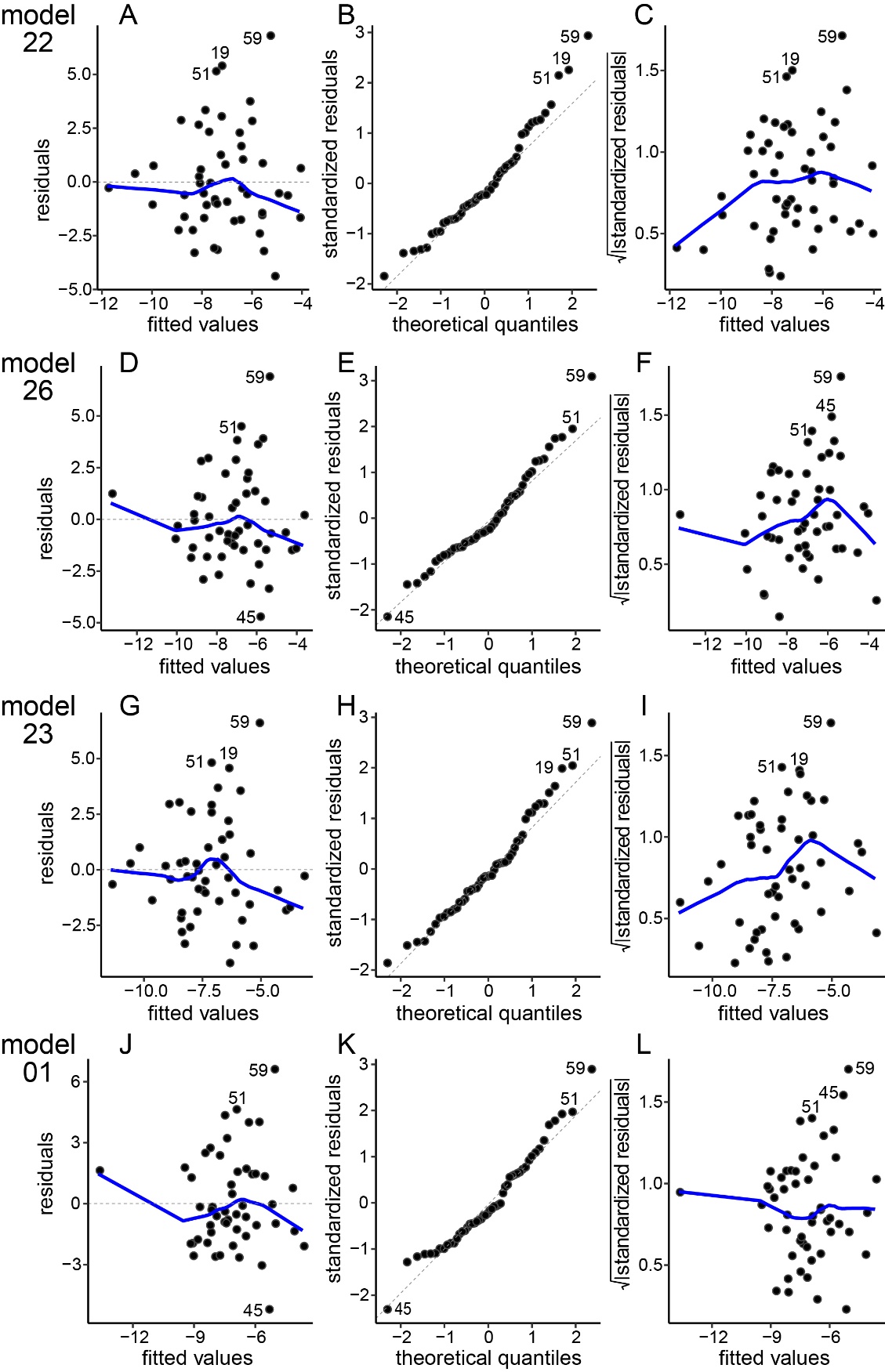


**Figure S3. Model diagnostic plots for the top (most parsimonious) models of annual changes (Δ) in traffic volume across states.** Diagnostic plots demonstrate that standard regression assumptions are met for all four of the top models: (A, D, G, J) residual vs. fitted plots demonstrate that assumptions of *linearity* are met; (B, E, H, K) normal Q-Q plots demonstrate that assumptions of *normality* are met; (C, F, I, L) scale-location plots demonstrate that assumptions of *homoscadescity* are met. Model numbers correspond to those in Table S3 and S4.
